# An examination of mediation by DNA methylation on birthweight differences induced by assisted reproductive technologies

**DOI:** 10.1186/s13148-022-01381-w

**Published:** 2022-11-28

**Authors:** Ellen Ø. Carlsen, Yunsung Lee, Per Magnus, Astanand Jugessur, Christian M. Page, Haakon E. Nustad, Siri E. Håberg, Rolv T. Lie, Maria C. Magnus

**Affiliations:** 1 Centre for Fertility and Health, Norwegian Institute of Public Health, Oslo, Norway; 2 Department of Community Medicine, Institute of Health and Society, University of Oslo, Oslo, Norway; 3 Department of Global Public Health and Primary Care, University of Bergen, Bergen, Norway; 4 Department of Mathematics, Faculty of Mathematics and Natural Sciences, University of Oslo, Oslo, Norway; 5 Deepinsight, Oslo, Norway

**Keywords:** Assisted reproductive technology, DNA methylation, Birthweight, MoBa, MBRN

## Abstract

**Background:**

Children born after assisted reproductive technologies (ART) differ in birthweight from those naturally conceived. It has been hypothesized that this might be explained by epigenetic mechanisms. We examined whether cord blood DNA methylation mediated the birthweight difference between 890 newborns conceived by ART (764 by fresh embryo transfer and 126 frozen thawed embryo transfer) and 983 naturally conceived newborns from the Norwegian Mother, Father, and Child Cohort Study (MoBa). DNA methylation was measured by the Illumina Infinium MethylationEPIC array. We conducted mediation analyses to assess whether differentially methylated CpGs mediated the differences in birthweight observed between: (1) fresh embryo transfer and natural conception and (2) frozen and fresh embryo transfer.

**Results:**

We observed a difference in birthweight between fresh embryo transfer and naturally conceived offspring of − 120 g. 44% (95% confidence interval [CI] 26% to 81%) of this difference in birthweight between fresh embryo transfer and naturally conceived offspring was explained by differences in methylation levels at four CpGs near *LOXL1*, *CDH20*, and *DRC1*. DNA methylation differences at two CpGs near *PTGS1* and *RASGRP4* jointly mediated 22% (95% CI 8.1% to 50.3%) of the birthweight differences between fresh and frozen embryo transfer.

**Conclusion:**

Our findings suggest that DNA methylation is an important mechanism in explaining birthweight differences according to the mode of conception. Further research should examine how gene regulation at these loci influences fetal growth.

**Supplementary Information:**

The online version contains supplementary material available at 10.1186/s13148-022-01381-w.

## Introduction

The birthweight of singleton newborns conceived using assisted reproductive technologies (ART) differs from naturally conceived newborns [1–3]. Interestingly, birthweight varies according to the use of embryo cryopreservation. Children born after fresh embryo transfer have lower birthweight, and children born after frozen embryo transfer have a slightly higher birthweight compared to natural conceptions [4–8]. The mechanisms explaining these birthweight differences remain elusive. The ART procedures themselves may directly impact the developing embryo [9, 10]. Furthermore, hormones used to induce ovulation may influence the intrauterine environment or specific characteristics of the growth medium used for embryo culture may alter fetal growth [1–3].

Another plausible mechanism may stem from differences in DNA methylation levels at specific cytosine-phosphate-guanine (CpG) sites in ART-conceived children versus naturally conceived children. ART procedures coincide with the periconceptional period when the early embryo undergoes extensive epigenetic reprogramming [11, 12], which potentially could perturb the process by which epigenetic marks are removed and a different set of DNA methylation marks is established. Previous studies report that cord blood DNA methylation varies according to the mode of conception [12–17], birthweight [18], and gestational age [19, 20]. The effects of smoking in pregnancy on birthweight have been proposed to be in part mediated by DNA methylation levels, but results are uncertain due to possible bias introduced by misclassification of the exposure [21–23].

Our primary objective was to examine whether cord blood DNA methylation mediated the difference in birthweight observed in fresh embryo transfer versus natural conception. As a secondary objective, we also compared frozen and fresh embryo transfer newborns. We used data from newborns in the Norwegian Mother, Father, and Child Cohort Study (MoBa) [16, 24], on 764 ART newborns conceived by fresh embryo transfer, 126 ART newborns conceived by frozen embryo transfer, and 983 newborns conceived naturally (of whom 20 were intrauterine inseminations).

## Material and methods

### Study population

MoBa recruited pregnant women and their partners across Norway around the 18th week of gestation between 1999 and 2008 [24, 25]. Approximately 95,000 mothers, 75,000 fathers (included from 2001 onwards) and 114,000 children were included in the study, comprising approximately 40% of invited women. The MoBa participants filled out a series of questionnaires during pregnancy and at multiple time points after delivery. The current study is based on version 12 of the quality-assured data files released for research in 2019. Peripheral blood samples were taken from mothers and fathers at the time of recruitment, while umbilical cord blood samples were collected from the children at birth [26, 27].

**Fig. 1:**
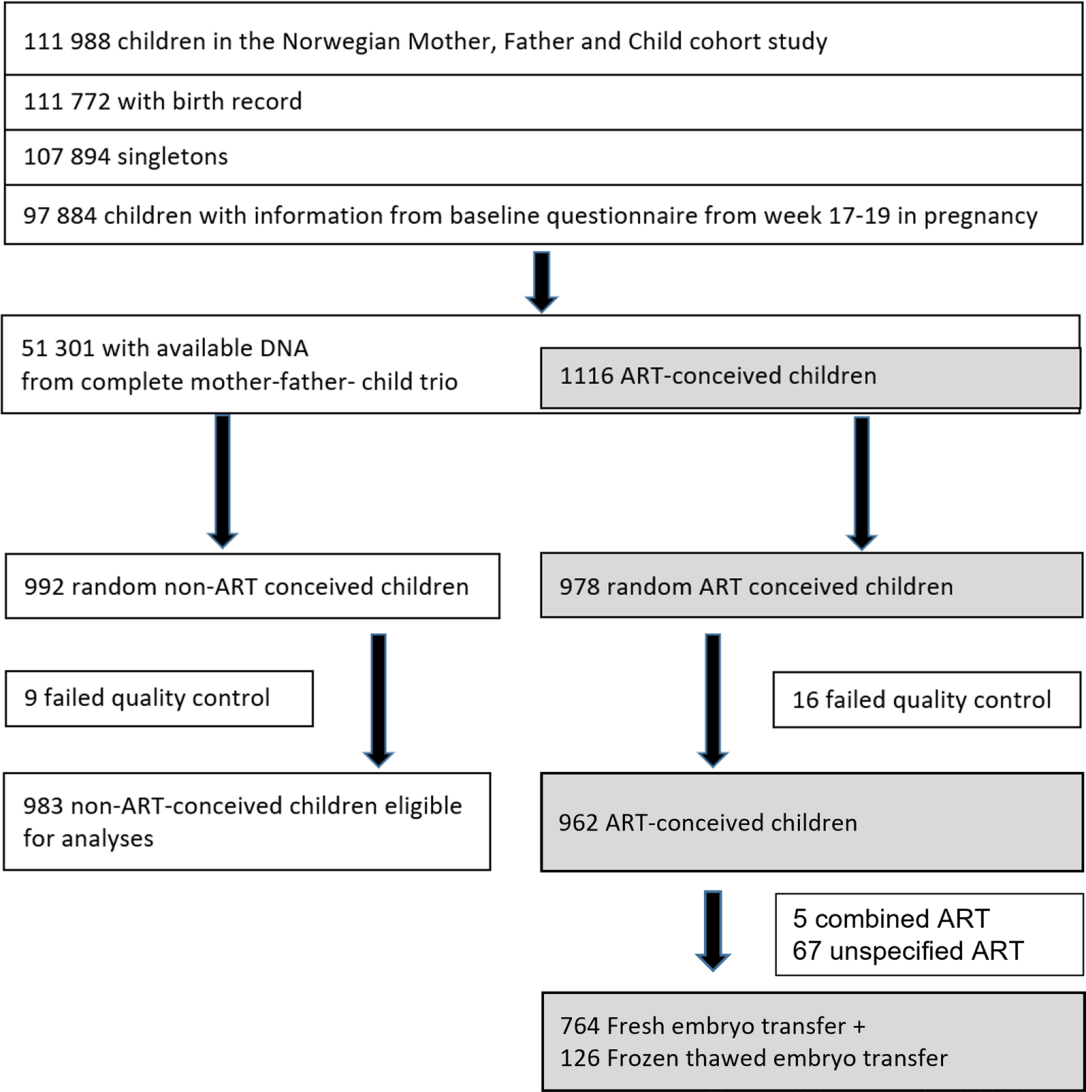
Selection of study participants

This study focused on a subset of mother–father–newborn trios in MoBa who met all the following criteria: 1) the children were singletons born between 2001 and 2009 with full records from the Medical Birth Registry of Norway, 2) the mothers had filled out the first MoBa questionnaire at the 18th week of gestation, and 3) the DNA samples from the complete trios were available. Among the trios who met all these criteria, we randomly selected 992 naturally conceived trios and all 978 ART trios (Fig. 1) [16].

This study was approved by the Regional Committees for Medical and Health Research Ethics of South/East Norway (#2017/1362). Participants in MoBa have provided informed consent. The establishment of MoBa and initial data collection was based on a license from the Norwegian Data Protection Agency and an approval from the Regional Committees for Medical and Health Research Ethics. The MoBa cohort is now regulated by the Norwegian Health Registry Act.

### Cord blood DNA methylation

DNA samples were analyzed at the Institute of Life & Brain Sciences at the University of Bonn in Germany. The EZ-96DNA Methylation-Lightning™MagPrep kit (Zymo Research, Irvine, USA) was used for bisulfite conversion. Cord blood DNA methylation of the 1,970 newborns was measured using the Illumina Infinium MethylationEPIC array (San Diego, CA, USA) [28].

Details of the quality control pipeline have been described [16]. Briefly, quality control was performed in four batches separately using the RnBeads R package [29]. We excluded 44,210 cross-hybridizing probes [30], 16,117 probes within three base pairs of SNPs, and probes with high detection *P* value (> 0.01). This resulted in 770,586 probes on the autosomes and 19,627 probes on the sex chromosomes. In this study, we only focused on the 770,586 autosomal probes. We excluded 25 newborns because of poor data quality; this included two newborns with empty plate wells, one with outlier values, three with corrupt images, and 19 with high background signals (Fig. 1). The fluorescence intensities were corrected for background noise using enmix.oob and normalized using the Beta-mixture quantile normalization [31] from the wateRmelon R package [32].

### ART and birthweight

Information on the use of ART, which is mandatory for fertility clinics to report, was obtained from the Medical Birth Registry of Norway. This included information on whether fresh or frozen embryo transfer was used. We excluded 72 ART newborns for whom the embryo transfer method was ambiguous, e.g., “combination of methods” or “unspecified” (Fig. 1). Intrauterine inseminations (*n* = 20) were included in the group of newborns conceived naturally, but excluded from the study population in a sensitivity analysis. Information on birthweight (in grams) was also obtained from the Medical Birth Registry of Norway.

### Potential confounders

A priori, we included maternal characteristics related to the use of ART associated with both cord blood DNA methylation levels and birthweight, as covariates. These included maternal age (continuous) [33], maternal smoking status during pregnancy (never, former, quit before the 18th week of gestation, or continued smoking after the 18th week of gestation) [34], maternal pre-pregnancy body mass index (BMI, continuous in kg/m^2^) [35], maternal educational level (less than high school, high school, up to four years of university, or more than four years of university) [36], and parity (strongly associated with the use of ART and birthweight [37]. We also adjusted for offspring sex (strongly associated with DNA methylation [38] and birthweight and thus a potential confounder of the mediator–outcome association) and the plate number used in the epigenome-wide analyses (to correct for batch effects). See Additional file 1: Fig. S1 for a schematic overview of the analysis. Finally, we adjusted for maternal intake of folic acid supplement (no intake or intake only before pregnancy, intake only during the first trimester, or intake both before and during the first trimester), as maternal folic acid supplement use is associated with DNA methylation levels in newborns [39] and birthweight [40, 41] and is therefore a potential confounder of the mediator–outcome relationship. As adjustment for gestational age may introduce biases [42, 43], this was not done in the main analysis.

### Statistical analyses

As a first step in the analysis, we identified differentially methylated CpGs between the newborns conceived naturally and those conceived by fresh embryo transfer. To do this, we regressed the transformed DNA methylation level, i.e., M value = log_2_ (Beta_value / (1-Beta_value)) [44], at each CpG on the use of fresh embryo transfer. We adjusted for maternal age, smoking status, pre-pregnancy BMI, parity, offspring sex, and plate number (as a random effect), using the rint.reg function from the Rfast package. In the second step of the analysis, we focused on the Bonferroni significant (*P* < 0.05) differentially methylated CpGs between the newborns conceived naturally and those conceived by fresh embryo transfer. We then regressed the transformed DNA methylation level on birthweight with adjustment for the same covariates as in the previous step in addition to maternal education and intake of folic acid supplement. (The lme function from the nlme package was used for this part of the analysis.) Again, the Bonferroni-corrected *P* < 0.05 was used to account for multiple testing.

The difference method [45] in a bootstrapping framework was used to estimate the indirect, direct, and total effect of fresh embryo transfer on birthweight through each of the fresh embryo transfer- and birthweight-associated CpGs. For each of 5,000 iterations, we estimated the total effect by regressing birthweight on the use of fresh embryo transfer and the covariates used in the second step as described above. We also estimated the direct effect by regressing birthweight on the use of fresh embryo transfer, one CpG at a time, and included the same covariates as above. Next, we estimated the indirect effect by subtracting the direct effect from the total effect. The corresponding 95% confidence intervals and *P* values for the indirect, direct, and total effects were obtained from the bootstrapped estimates.

We used the same approach to examine whether cord blood DNA methylation explained the differences in birthweight between fresh and frozen embryo transfer ART newborns. As the number of frozen embryo transfer children available for analysis was low (*n* = 126), we applied the Benjamini and Hochberg [46] procedure to control for multiple testing at a false discovery rate (FDR) < 0.05.

As we did not detect a significant birthweight difference between frozen transfer and naturally conceived newborns in our study, we were unable to perform a mediation analysis of birthweight differences.

### Sensitivity analyses

As cord blood cell-type composition could differ by mode of conception and birthweight, we performed a sensitivity analysis where we adjusted for cell-type composition.

As both DNA methylation levels and the birthweight of the newborn potentially could be affected by hormonal stimulation prior to an intrauterine insemination, we performed a sensitivity analysis excluding these newborns from the naturally conceived group. We performed the same steps as for the main analysis comparing naturally conceived newborns and those conceived by fresh embryo transfer.

Furthermore, as both birthweight and DNA methylation are highly correlated with gestational age [47], we conducted a sensitivity analysis investigating whether gestational age impacted the findings between birthweight and DNA methylation levels between newborns conceived naturally and by fresh embryo transfer. We performed an analysis of the birthweight-for-gestational age and sex, referred to as “birthweight Z-score” hereafter, using the same approach as in the main analysis. Here, we explored whether there was a difference in Z-score, whether any CpGs was associated with the Z-score, and whether these CpGs mediated any of the observed difference.

To assess the implications of the inflation factor in the EWAS of ART, we conducted additional sensitivity analysis applying a Bayesian method on the t statistics resulting from the EWAS of newborns conceived naturally and by fresh embryo transfer using the BACON package in R [48].

## Results

### Birthweight differences according to the mode of conception

Of the 1,873 newborns with DNA methylation data, 983 were conceived naturally, 764 were conceived using fresh embryo transfer, and 126 were conceived using frozen embryo transfer (Fig. 1). As reported elsewhere [1–3], we found that newborns conceived by fresh embryo transfer have a lower mean birthweight (− 159 g, Table 1) than those conceived naturally. A difference in birthweight between these two groups persisted after adjustment for maternal age, education, smoking status, pre-pregnancy BMI, parity, child’s sex, and maternal intake of folic acid (− 120 g, 95% confidence interval (CI) − 179, − 61, Table 2).

**Table 1:** Characteristics of study participants

Characteristics	Naturally conceived newborns (*n* = 983)	ART-conceived newborns—fresh embryo (*n* = 764)	ART-conceived newborns—frozen embryo (*n* = 126)
Maternal age at delivery, mean (SD)	30 (4.6)	33 (3.7)	33.6 (3.5)
Maternal parity, *N* (%)			
Nulliparous	461 (46.9%)	541 (70.8%)	78 (61.9%)
Multiparous	522 (53.1%)	223 (29.2%)	48 (38.1%)
Maternal educational level, *N* (%)			
Less than high school	71 (7.2%)	43 (5.6%)	3 (2.4%)
High school	299 (30.4%)	180 (23.6%)	31 (24.6%)
Up to 4 years of college	386 (39.3%)	334 (43.7%)	54 (42.9%)
More than 4 years of college	225 (22.9%)	204 (26.7%)	38 (30.2%)
Missing	2 (0.2%)	3 (0.4%)	0 (0%)
Maternal pre-pregnancy BMI, mean (SD)	24.3 (4.5)	24.4 (4.2)	23.8 (3.2)
Missing, *N* (%)	14 (1.4%)	15 (2%)	1 (0.8%)
Maternal smoking status during pregnancy, *N* (%)			
Never	490 (49.8%)	397 (52%)	65 (51.6%)
Former	253 (25.7%)	279 (36.5%)	47 (37.3%)
Quit before 18 gestational weeks	132 (13.4%)	47 (6.2%)	10 (7.9%)
Continued after 18 gestational weeks	104 (10.6%)	37 (4.8%)	4 (3.2%)
Missing	4 (0.4%)	4 (0.5%)	0 (0%)
Child sex, *N* (%)			
Male	470 (47.8%)	403 (52.7%)	66 (52.4%)
Female	513 (52.2%)	361 (47.3%)	60 (47.6%)
Child birthweight (grams), mean (SD)	3649.4 (525.6)	3490.3 (531.4)	3697.4 (577.2)
Missing, *N* (%)	0 (0%)	1 (0.1%)	0 (0%)
Maternal intake of folic acid supplement, *N* (%)			
Never or only before pregnancy	156 (15.9%)	49 (6.4%)	9 (7.1%)
In pregnancy before week 18	395 (40.2%)	84 (11%)	19 (15.1%)
Before and up to week 18 of pregnancy	432 (43.9%)	631 (82.6%)	98 (77.8%)

**Table 2:** Effects of DNA methylation on birthweight differences between fresh and naturally conceived newborns

	Total effect^1, 2^	*P* value						
Naturally conceived (reference) vs fresh embryo transfer	− 120 (− 179, − 61)	6.35E−05						

CpG name	Indirect effect^1, 2^ in grams	*P* value^1^	Direct effect^1, 2^ in grams	*P* value	Mediation proportion	CHR^3^	MAPINFO^3^	UCSC RefGene Name^3^
cg10372921	− 23 (− 34, − 13)	3.49E−05	− 98 (− 156, − 38)	1.22E−03	20.4%	15	74,218,733	*LOXL1*^4^
cg25423077	− 16 (− 27, − 8)	1.04E−03	− 104 (− 163, − 45)	5.85E−04	14.5%	18	59,221,601	*CDH20*^5^
cg02050426	− 15 (− 26, − 7)	1.41E−03	− 105 (− 164, − 46)	4.94E−04	13.5%	18	59,221,458	*CDH20*^5^
cg15138396	− 15 (− 27, − 6)	3.98E−03	− 105 (− 165, − 44)	5.86E−04	13.6%	2	26,624,450	*DRC1*^6^

	Combined indirect effect^1, 2^	*P* value^1^	Combined direct effect^1, 2^	*P* value	Mediation proportion			
The four CpGs combined	− 53 (− 71, − 37)	9.50E−10	− 67 (− 125, − 6)	2.86E−02	44.1%			

^1^Adjusted for maternal age, education, smoking status, pre-pregnancy BMI, parity, child’s sex, maternal intake of folic acid, and plate number. Birthweight difference is in grams

^2^95% confidence intervals in parenthesis

^3^Derived from the Illumina MethylationEPIC v1.0 B5 manifest file. The archaic names of the genes were updated based on the data from https://www.genenames.org/

^4^Located within 0–200 bases upstream of the transcription start site of the gene

^5^Located within the gene body

^6^Located within 200–1500 bases upstream of the transcription start site of the gene

### Mediation by cord blood DNA methylation in birthweight differences according to the mode of conception

**Fig. 2:**
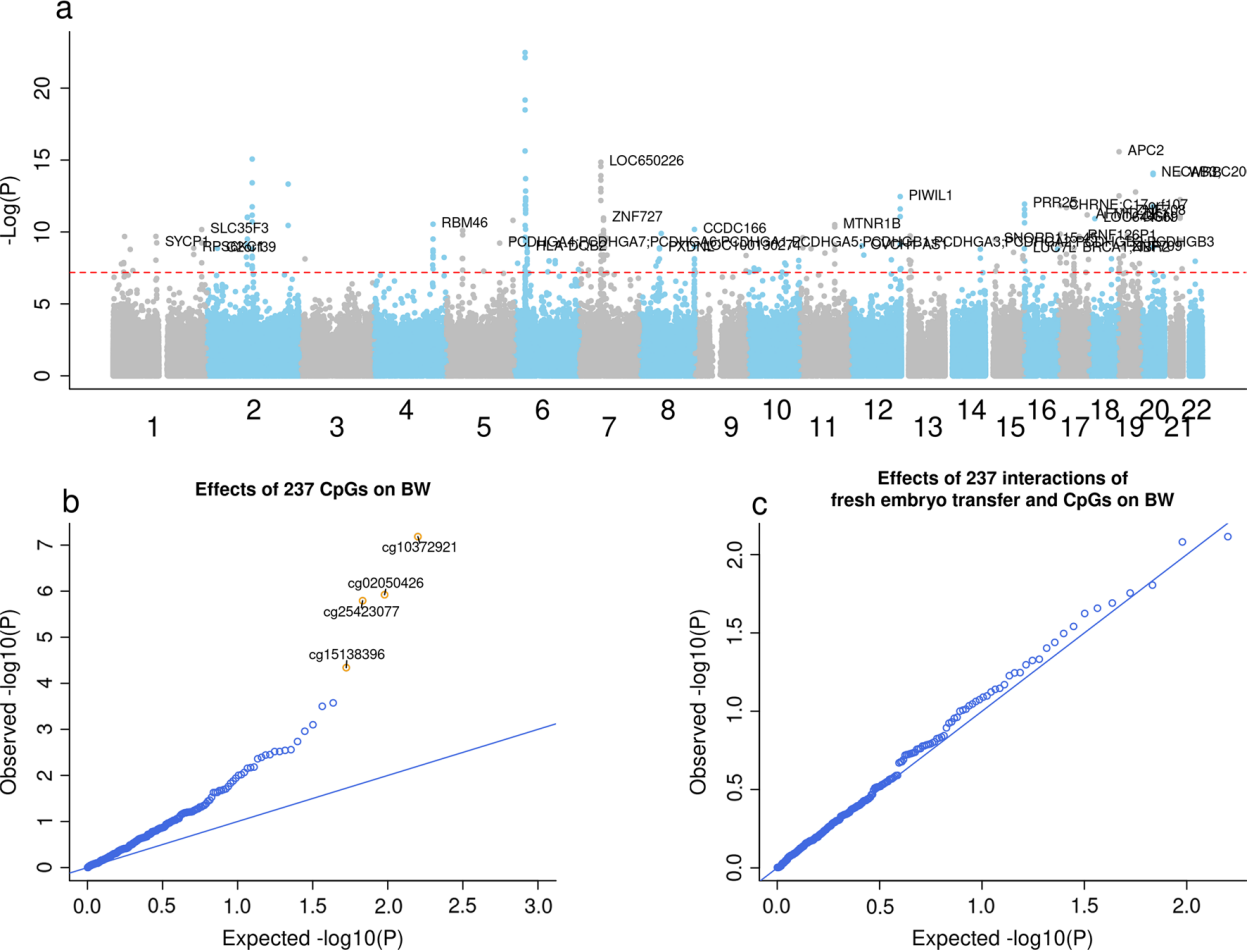
Differentially methylated CpGs between newborns conceived naturally and by fresh embryo transfer and birthweight-associated CpGs. **a** Manhattan plot displaying the 237 differentially methylated CpGs between newborns conceived naturally and those conceived by fresh embryo transfer. The red dotted line refers to the Bonferroni threshold (*P* = 0.05/770,564). Adjustment variables include maternal age, smoking status, pre-pregnancy BMI, parity, offspring sex, and plate number. **b** Quantile–quantile plot showing the birthweight-associated CpGs among the 237 fresh embryo transfer-associated CpGs. The yellow dots refer to the CpGs that were also associated with birthweight. Adjustment variables include those mentioned in (**a**) and maternal education and intake of folic acid. **c** Quantile–quantile plot showing the interactions of fresh embryo transfer and CpGs on birthweight. Adjustment variables were those mentioned in (**b**)

We identified 237 differentially methylated CpGs between naturally conceived and fresh embryo transfer conceived newborns after adjusting for maternal age, smoking status, pre-pregnancy BMI, parity, newborn sex, and plate (Bonferroni-corrected *P* < 0.05, equivalent to *P* < 6.49E−08, Fig. 2a) (Additional file 8) ﻿[16]. This analysis showed an inflation factor of 2.07. We searched for associations between the DNA methylation level at each of these 237 associated CpGs and birthweight. We detected four significant CpGs (Bonferroni-corrected *P* < 2.11E−04, Fig. 2b and Table 2). We also tested for interaction effects between each of the 237 CpGs (“exposure–mediator interaction”) on birthweight because the presence of interaction effects was a decisive factor in choosing which methodology to use for the current mediation analyses [49]. As we did not observe any evidence of significant interaction effects of fresh embryo transfer and DNA methylation at these CpGs on birthweight (Fig. 2c), we proceeded with the mediation analysis using the difference approach as described in Baron and Kenny, 1986 [45] (Table 2).

This approach compares the effect size of fresh embryo transfer on birthweight with and without adjustment for potential mediators [50]. One of the CpGs, cg10372921, located near the lysyl oxidase like 1 (*LOXL1*) gene, mediated the effect of fresh embryo transfer on birthweight by 20% (− 23 g, 95% CI − 34, − 13; *P* = 3.49E−05). The two CpGs, cg25423077 and cg02050426, located within the cadherin 20 (*CDH20*) gene, mediated the association by 15% (− 16 g, 95% CI − 27, − 8; *P* = 1.04E−03) and 14% (− 15 g, 95% CI − 26, − 7; *P* = 1.41E−03), respectively. We note that the indirect effects through these two CpGs were similar most likely because they are located near each other in the genome (Pearson correlation coefficient = 0.88, Additional file 2: Fig. S2). The last CpG, cg15138396, located near the dynein regulatory complex subunit 1 (*DRC1*) gene, mediated the association by 14% (− 15 g, 95% CI − 27, − 6; *P* = 3.98E−03). The overall proportion mediated by the four CpGs together was 44% (95% CI 26%, 81%).

### Sensitivity analyses

In the sensitivity analysis where we also adjusted for cell-type composition in cord blood, we identified 270 differentially methylated CpGs (Additional file 3: Fig. S3). Compared to the main analysis, seven additional CpGs to three of the four CpGs detected in the main analysis were found to be associated with birthweight (Additional file 4: Table S1). Together, the overall proportion of the difference in birthweight mediated by these ten CpGs was 54.7%.

**Fig. 3:**
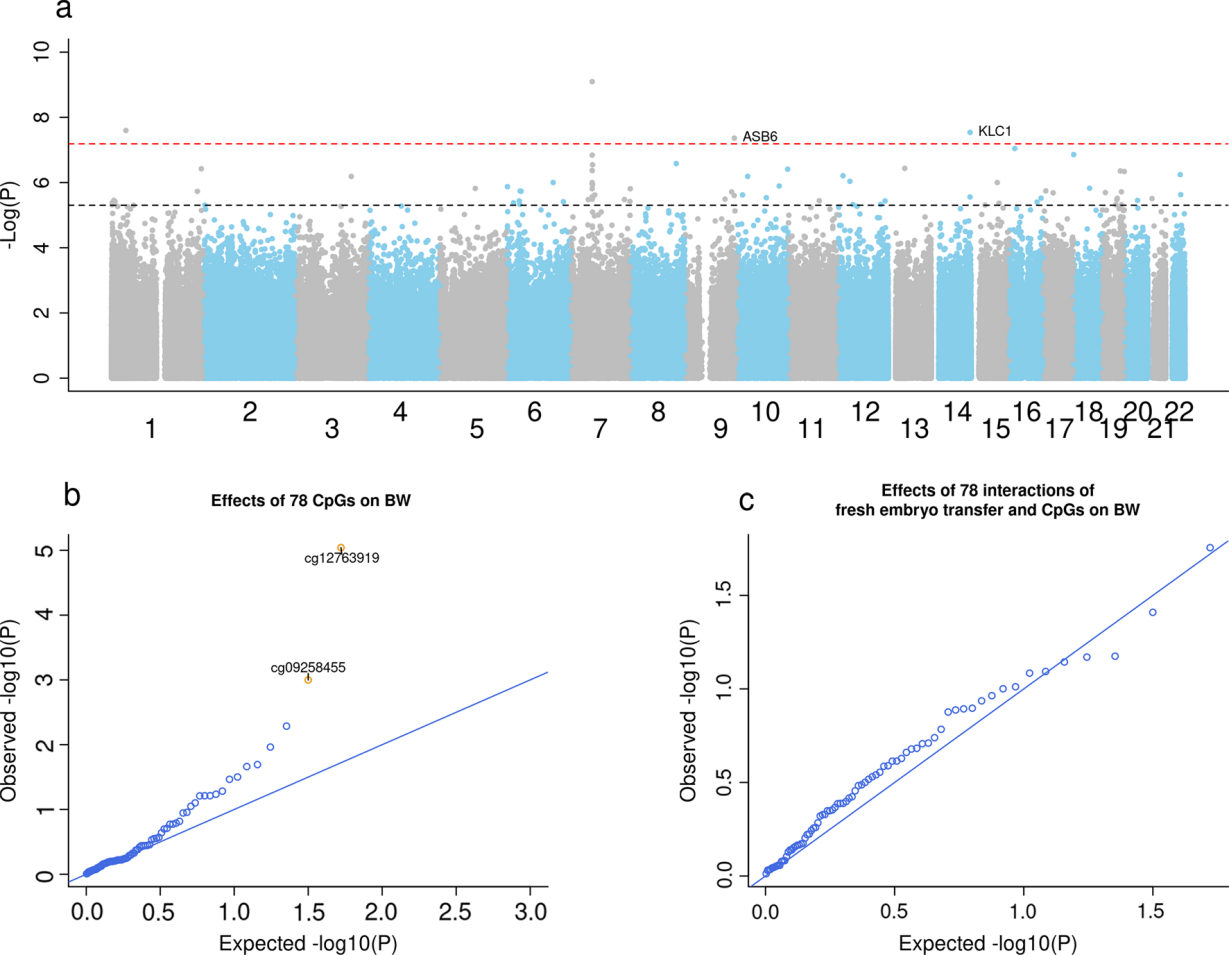
Differentially methylated CpGs between newborns conceived by fresh and frozen embryo transfer and birthweight-associated CpGs. **a** Manhattan plot displaying the 78 differentially methylated CpGs (*P*_FDR_ < 0.05) between the newborns conceived by fresh embryo transfer (*n* = 764) and frozen thawed embryo transfer (*n* = 126). The red dotted line refers to the Bonferroni threshold (*P* = 0.05/770,564), whereas the other dotted line in black refers to the FDR threshold. Adjustment variables include maternal age, smoking status, pre-pregnancy BMI, parity, offspring sex, and plate number. **b** Quantile–quantile plot showing the birthweight-associated CpGs among the 78 frozen thawed embryo transfer-associated CpGs. The yellow dots refer to the CpGs that were also associated with birthweight. Adjustment variables include those mentioned in (**a**) and maternal education and intake of folic acid supplement. **c** Quantile–quantile plot showing the interactions of frozen thawed embryo transfer (fresh embryo transfer as a reference) and CpGs on birthweight. Adjustment variables were those mentioned in (**b**)

In the sensitivity analysis in which 20 newborns conceived by intrauterine inseminations were excluded from the comparison group (those conceived naturally), we identified 270 differentially methylated CpGs after adjusting for the same covariates as in the main analysis (Additional file 5: Fig. S4). In addition to the four CpGs found in the main analysis, additionally three CpGs were found to be significantly associated with birthweight (Additional file 4: Table S2). Together, the overall proportion of the difference in birthweight mediated by these seven CpGs was 64.3% (95% CI 39.7%, 94.3%). The newborns conceived using fresh embryo transfer had lower birthweight Z-score than those conceived naturally (− 0.22, 95% CI − 0.33, − 0.11), and 34.2% of the difference in birthweight Z-score was mediated by 11 CpG sites (Additional file 4: Table S3 and Additional File 6: Fig. S5).

**Table 3:** Effects of DNA methylation on birthweight differences between fresh and frozen conceived newborns

	Total effect^1, 2^	*P* value						
Fresh embryo transfer (reference) vs frozen thawed embryo transfer	194 (89, 301)	3.22E−04						

CpG name	Indirect effect^1, 2^ in grams	*P* value^1^	Direct effect^1, 2^ in grams	*P* value	Mediation proportion	CHR^3^	MAPINFO^3^	UCSC RefGene Name^3^
cg12763919	35 (13,65)	7.96E−03	159 (55, 263)	2.74E−03	19.3%	9	125,137,580	*PTGS1*^4^
cg09258455	24 (6,47)	2.31E−02	170 (65, 276)	1.56E−03	13.2%	19	38,918,162	*RASGRP4*^5^

	Combined indirect effect^1, 2^	*P* value^1, 2^	Combined direct effect^1, 2^	*P* value	Mediation proportion			
The two CpGs combined	42 (16, 75)	4.71E−03	152 (47, 256)	4.30E−03	21.6%			

^1^Adjusted for maternal age, education, smoking status, pre-pregnancy BMI, parity, child’s sex, maternal intake of folic acid, and plate. Birthweight difference is in grams

^2^95% confidence intervals in parenthesis

^3^Derived from the Illumina MethylationEPIC v1.0 B5 manifest file. The archaic names of the genes were updated based on the data from https://www.genenames.org/

^4^Located within the 5’ untranslated region, 0–200 bases upstream of the transcription start site and the gene body depending on the transcript

^5^Located within 200–1500 bases upstream of the transcription start site of the gene

In the sensitivity analysis where we applied the BACON method to adjust for the inflation factor from the EWAS, the inflation factor decreased to 1.33 and the number of significant ART-associated CpGs was 234 (Additional File 7: Fig. S6). Compared to the main analysis, two additional CpGs, in addition to three of the four CpGs in the main analysis, were found to be associated with birthweight (Additional file 4: Table S4). Together, these five CpGs mediated 28.4% of the birthweight difference.

### Mediation by cord blood DNA methylation in birthweight differences between fresh and frozen embryo transfer offspring

The adjusted mean birthweight of the ART newborns conceived using fresh embryo transfer (*n* = 764) was 194 g lower than that of the ART newborns conceived using frozen embryo transfer (*n* = 126) (Table 3). When comparing these two ART groups, we identified 78 differentially methylated CpGs (*P*_FDR_ < 0.05, Fig. 3a) (Additional file 9) [51]. This analysis showed an inflation factor of 1.85. Two of the 78 CpGs were also associated with birthweight (*P*_FDR_ < 0.05, Fig. 3b). As shown in Fig. 3c, we found no evidence of significant interactions of frozen embryo transfer (fresh embryo transfer as a reference) and CpGs. Two CpGs, cg12763919, near the gene prostaglandin endoperoxide synthase 1 (*PTGS1*), and cg09258455, near the gene RAS guanyl-releasing protein 4 (*RASGRP4*), mediated the birthweight differences between fresh and frozen embryo transfer newborns by 19% (35 g, 95% CI 13, 65, *P* = 7.96E−03, Table 3) and 13% (24 g, 95% CI 6, 47, *P* = 2.31E−02), respectively. The two CpGs combined mediated 22% of the difference in birthweight (95% CI 8.1%, 50.3%).

## Discussion

Our main aim was to investigate whether differences in DNA methylation explained the previously reported differences in birthweight according to the mode of conception. We found that cord blood DNA methylation levels at four CpG sites explained 44% of the difference in birthweight between newborns conceived with fresh embryo transfer and those conceived naturally. Further, cord blood methylation differences at two CpG sites explained 22% of the difference in birthweight between newborns conceived with fresh embryo transfer and those conceived with frozen embryo transfer.

The observed magnitude of the difference in birthweight in our study between the newborns conceived using fresh embryo transfer and those conceived naturally is consistent with previous findings [1–3]. Furthermore, the observation that the newborns conceived using frozen embryo transfer are heavier than those conceived using fresh embryo transfer has also been reported previously [4–8].

For fresh embryo transfer, one of the differentially methylated CpGs that mediated the difference in birthweight was located near *LOXL1*. *LOXL1* encodes a member of the lysyl oxidase family of proteins, and its expression has been associated with premature rupture of membranes (PROM) [52, 53]. *LOXL1* has also been linked to birthweight in a previous EWAS of birthweight [18]. A plausible explanation for this association is that the decreased methylation level in ART-conceived offspring near this gene may increase the risk of PROM, resulting in lower gestational age and thereby lower birthweight. We did not assess whether this CpG was related to gestational age in our analysis. But given that fresh embryo transfer newborns generally have a shorter gestational age and an increased risk of PROM [2], and the effects of birthweight and gestational age are difficult to disentangle, a plausible mechanism could be through gestational age. We, therefore, conducted a sensitivity analysis of birthweight Z-score, although any adjustment for gestational age when analyzing differences in birthweight is inherently problematic and should be interpreted with caution [42, 43]. Differences in DNA methylation levels at 11 CpG sites mediated 34% of the differences in birthweight Z-score. The reduction in the absolute level of mediated proportion by DNA methylation when standardizing for gestational age (34% compared to 44% in the main analysis) may imply that part of the mediation pathway from the mode of conception to birthweight occurs via factors affecting the timing of birth, although bias introduced by including gestational age in the analysis cannot be excluded. This included the CpGs cg10372921 located near *LOXL1* and cg25423077 and cg02050426 located near *CDH20*. This can imply that cytosine methylation at the three CpG sites has an impact on not only birthweight but also fetal growth velocity. The same CpG sites were also included in the sensitivity analysis also adjusting for cell-type composition, where we found that differences in DNA methylation levels at 10 CpG sites mediated 55% of the difference in birthweight.

*CDH20* belongs to the cadherin superfamily of genes and is one of three cadherin 7-like genes. Gain- and loss-of-function analyses in animal models have demonstrated a pivotal role of cadherins in several key cellular processes, including neural patterning, cell migration, axon guidance, synapse formation, and synapse function [54–56]. Given the wide range of functions of the cadherin superfamily, the precise mechanism for how DNA methylation of *CDH20* could influence birthweight is unclear. However, this gene was also found in a previous EWAS of birthweight [18]. A few studies have shown that children conceived using ART differ in neurological development compared to those conceived naturally [57]. Our findings underscore the need for further investigations into whether DNA methylation in *CDH20* might partly explain some of the differences in neurodevelopment between these two groups of children.

*DRC1* encodes a central component of the nexin–dynein complex (N-DRC). This gene appears to be expressed in decidual cells and is thought to influence the maternal–fetal immune relationship [58, 59]. We could not find evidence in the literature linking *DRC1* expression to fetal growth.

In the sensitivity analysis in which we also adjusted for cell-type composition, an additional three CpGs in “PARN Like Ribonuclease Domain Containing Exonuclease 1” (*PNLDC1*) and one CpG site in the 5’ untranslated region of “RNA Binding Motif Protein 46” (*RBM46*) mediated a proportion of the birthweight difference. Both *PNLDC1* and *RBM46* are involved in gonadal development and spermatogenesis and are mainly expressed in testis [60–63], but we could not find any evidence in the literature linking these two genes to fetal growth.

In the sensitivity analysis in which we excluded infants conceived by intrauterine inseminations from the control group, a CpG located near the gene Amyloid Beta Precursor Protein Binding Family A Member 1 (*APBA1*), mediated a proportion of the difference in birthweight (in addition to the already mentioned CpGs). This gene is expressed in the brain [64] and is involved in the vulval development of Caenorhabditis elegans [65], but we could not find evidence that it is linked to fetal growth.

We identified two more CpG sites, one in *PTGS1* and the other in *RASGRP4*, that mediated the birthweight differences between fresh and frozen embryo transfer newborns. As we used a lower statistical significance threshold in this secondary analysis, the results need to be validated in other studies.

Prostaglandin (PG) H synthase exists as two isoforms, also known as cyclooxygenases (COX-1 and COX-2), which are encoded by *PTGS1* and *PTGS2*, respectively. In mice, a malfunctioning *Ptgs2* results in multiple reproductive failures [66, 67], whereas *Ptgs1*-deficient mice exhibit normal fertility, but delayed labor and fewer live offspring [68, 69]. However, the exact role of this gene in both labor failure and intrauterine growth remains unclear, but the gene was found to be associated with birthweight in a large previously published EWAS of birthweight [18]. The protein encoded by *RASGRP4* is a member of the Ras guanyl nucleotide-releasing protein family of Ras guanine nucleotide exchange factor. *RASGRP4* is expressed in lymphoma and leukemia [70–72]. Increased birthweight has been linked to increased risk of childhood cancers [73], which is interesting in the context of increased birthweight in frozen thawed embryo transfer newborns and their increased risk of childhood cancer [74]. However, if this is related to differential methylation of *RASGRP4* in frozen thawed and fresh embryo transfer newborns remains to be elucidated. Furthermore, the mechanism through which the altered DNA methylation influencing fetal growth is related to the catch-up growth seen in ART-conceived children [75] and possibly later risk of disease is still unknown. Combined with reports indicating that children conceived by frozen thawed embryo transfer show an increased risk of childhood cancers [74, 76, 77], future studies are warranted to examine this cancer link more closely and determine how *RASGRP4* might contribute to the difference in birthweight between newborns conceived using fresh versus frozen embryo transfer.

Although the participants in this study stem from a nationwide pregnancy cohort, they are not completely representative of Norwegian births in the general population due to self-selection into the MoBa cohort [78]. Furthermore, socio-demographic variables of the parents who use ART differ from those who do not use ART [79]. While we used multivariate adjustment to account for these differences, we cannot rule out the possibility of residual confounding. Residual confounding is of particular concern in the interpretation of mediation analyses because we need to account for not only confounders of the “exposure–outcome” relationship (ART and birthweight), but also the “exposure–mediator” relationship (ART and DNA methylation), in addition to the “mediator–outcome” relationship (DNA methylation and birthweight) [80].

The high inflation factor we observed in the EWAS of ART is likely due to a global shift in the methylation levels [16] or unmeasured confounding. Batch effects are not particularly likely since ART and non-ART samples were randomly allocated to different plates and batches. The inflation could be observed when an exposure of interest has a small effect on many genomic loci [20, 48, 81]. However, after correcting for the inflation factor in a sensitivity analysis applying the BACON software [48], five CpGs mediated 28% of the difference in birthweight between non-ART and ART fresh conceived newborns, compared to 44% in the main analysis. The reduction in total mediated proportion was probably induced by losing the CpG cite in *LOXL1* after applying the correction, which was the CpG most strongly associated with birthweight in the main analysis.

The use of cord blood to examine differences in DNA methylation levels that impact birthweight can only act as a surrogate tissue for assessing fetal growth in utero. It is likely that there are mechanisms related to fetal growth that are not adequately captured by cord blood but that might be captured more explicitly by placental tissue.

Furthermore, future studies with larger sample sizes and/or using different methods, such as examining mediation through differentially methylated regions instead of single CpG sites, may expand on understanding the biological pathways from mode of conception through DNA methylation on fetal growth.

Since both the measurement of birthweight and the collection of cord blood samples were conducted at the time of birth, the casual direction should be interpreted with caution. As the bulk of epigenetic reprogramming occurs at conception and the major contribution to birthweight happens at the end of pregnancy [11, 82], it is less likely that birthweight influences the observed DNA methylation level differences than the opposite. Additionally, adjustment for birthweight in the previously published EWAS of ART [16] did not change the results significantly, suggesting that birthweight is not an important explanatory factor of the association between mode of conception and cord blood DNA methylation levels.

Furthermore, there is some evidence in the literature showing that differences in DNA methylation levels in blood according to the mode of conception wane over time with increasing age of the offspring [13, 83], which could indicate that DNA methylation differences at birth are related to birth outcomes and not necessarily later health, though this hypothesis needs to be tested in larger studies.

## Conclusions

Our study supports that cord blood DNA methylation contributes to birthweight differences according to the mode of conception. The exact mechanisms for how these methylation differences impact on intrauterine growth warrant further investigation and could be clinically important for couples who resort to ART to conceive.

## Supplementary Information

** Additional file 1: Fig. S1**. Directed acyclic graph of our analysis. BW; birthweight. C1; confounder of the association between ART and DNA methylation at CpG sites. C2: confounder of the association between the CpG sites and birthweight. C3: confounder of the association between ART and birthweight.

**Additional file 2: Fig. S2**. Pearson correlation coefficients among the four CpGs mediated between ART (naturally conceived vs fresh embryo transfer) and birthweight.

**Additional file 3: Fig. S3**. Main analysis with additional adjustment for cell-type composition. (a) Manhattan plot displaying the 256 differentially methylated CpGs between newborns conceived naturally and those conceived by fresh embryo transfer. The red dotted line refers to the Bonferroni threshold (P = 0.05/770,564). Adjustment variables include maternal age, smoking status, pre-pregnancy BMI, parity, offspring sex, plate number and cell-type composition. (b) Quantile–quantile plot showing the birthweight-associated CpGs among the 256 fresh embryo transfer-associated CpGs. The yellow dots refer to the CpGs that were also associated with birthweight. Adjustment variables include those mentioned in (a) and maternal education and intake of folic acid. (c) Quantile–quantile plot showing the interactions of fresh embryo transfer and CpGs on birthweight. Adjustment variables were those mentioned in (b).

**Additional file 4: Table S1**. Mediation analyses of the birthweight differences between fresh embryo transfer conceived newborns and naturally conceived newborns after additional adjustment for cell-type composition in cord blood. This file contains **S-Table 2**: mediation analyses of the birthweight differences between fresh embryo transfer conceived newborns and naturally conceived newborns after excluding newborns conceived by intrauterine inseminations from the control group. This file contains **S-Table 3**: mediation analyses of the growth differences between fresh embryo transfer conceived newborns and naturally conceived newborns now using birthweight Z-score instead of birthweight. This file contains **S-Table 4**: mediation analyses of the growth differences between fresh embryo transfer conceived newborns and naturally conceived newborns after correction for inflation factor in the EWAS using the BACON method.

**Additional file 5: Fig. S4**. Main analysis excluding 20 cases of intrauterine insemination. (a) Manhattan plot displaying the 270 differentially methylated CpGs between newborns conceived naturally (n = 963; the cases of insemination were excluded) and those conceived by fresh embryo transfer (n = 764). The red dotted line refers to the Bonferroni threshold (P = 0.05/770,564). Adjusting variables include maternal age, smoking status, pre-pregnancy BMI, parity, offspring sex, and plate number. (b) Quantile–quantile plot showing the birthweight-associated CpGs among the 270 fresh embryo transfer-associated CpGs. The yellow dots refer to the CpGs that were also associated with birthweight. Adjusting variables include those mentioned in (a) and maternal education and intake of folic acid. (c) Quantile–quantile plot showing the interactions of fresh embryo transfer and CpGs on birthweight. Adjusting variables were those mentioned in (b).

**Additional file 6: Fig. S5**. Differentially methylated CpGs between newborns conceived naturally and by fresh embryo transfer and Z-score-associated CpGs. (a) Quantile–quantile plot showing the Z-score-associated CpGs among the 237 fresh embryo transfer-associated CpGs. The yellow dots refer to the CpGs that were also associated with Z-score. (b) Quantile–quantile plot showing the interactions of fresh embryo transfer and CpGs on Z-score.

**Additional file 7: Fig. S6**. Differentially methylated CpGs between newborns conceived naturally and by fresh embryo transfer and birthweight-associated CpGs after applying BACON correction for inflation. (a) Quantile–quantile plot showing the birthweight-associated CpGs among the 234 fresh embryo transfer-associated CpGs. The yellow dots refer to the CpGs that were also associated with birthweight. (b) Quantile–quantile plot showing the interactions of fresh embryo transfer and CpGs on birthweight.

**Additional file 8**. This file contains the summary statistics from 1) the EWAS of ART (the newborns conceived naturally versus those conceived by fresh embryo transfer; see the columns with the suffix of “_ART”), 2) the association analysis between the fresh embryo transfer-associated CpG and birthweight (see the columns with the suffix of “_BW”), and 3) the statistical test of the interaction terms of fresh embryo transfer and CpGs on birthweight (see the columns of the suffix of “ART_CPG_inter”). b: estimated beta coefficient, se: estimated standard error, df: degree of freedom, n0: the number of controls, n1: the number of cases, t: T statistic, p: P value, q: Q value, i.e., adjusted P value using the Benjamini and Hochberg ^46^ procedure.

**Additional file 9**. This file contains the summary statistics from 1) the EWAS of ART (fresh versus frozen embryo transfer; see the columns with the suffix of “_ART”), 2) the association analysis between the frozen embryo transfer-associated CpG and birthweight (see the columns with the suffix of “_BW”), and 3) the statistical test of the interaction terms of frozen embryo transfer and CpGs on birthweight (see the columns of the suffix of “ART_CPG_inter”). b: estimated beta coefficient, se: estimated standard error, df: degree of freedom, n0: the number of controls, n1: the number of cases, t: T statistic, p: P value, q: Q value, i.e., adjusted P value using the Benjamini and Hochberg ^46^ procedure.

## Data Availability

The data that support the findings of this study are available from NIPH, but restrictions apply regarding the availability of these data. Access can be obtained by applying to NIPH at https://www.fhi.no/en/studies/moba/. Access can only be given after approval by the Norwegian Ethical committees on the grounds that the applications are consistent with the consent provided. Specific questions regarding access for data in this study can be directed to Yunsung.Lee@fhi.no.
