# Supplementary figures and images for: An examination of mediation by DNA methylation on birthweight differences induced by assisted reproductive technologies
Source: Clin Epigenetics. 2022 Nov 28;14:151. doi: 10.1186/s13148-022-01381-w (PMC9703677; doi:10.1186/s13148-022-01381-w)

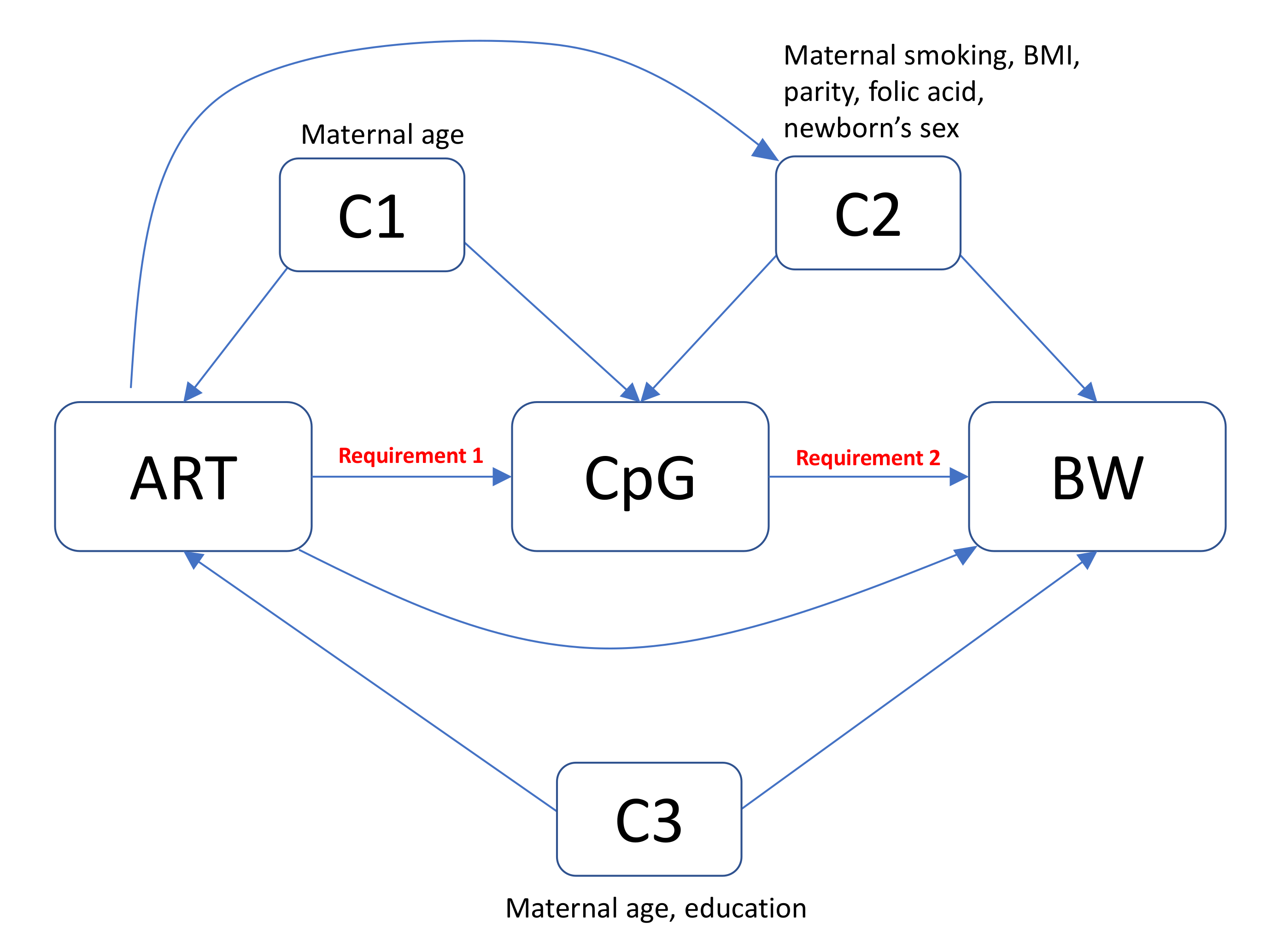

Supplement: Supplementary file 1 — Additional file 1: Fig. S1. Directed acyclic graph of our analysis. BW; birthweight. C1; confounder of the association between ART and DNA methylation at CpG sites. C2: confounder of the association between the CpG sites and birthweight. C3: confounder of the association between ART and birthweight. [file 13148_2022_1381_MOESM1_ESM.tif]

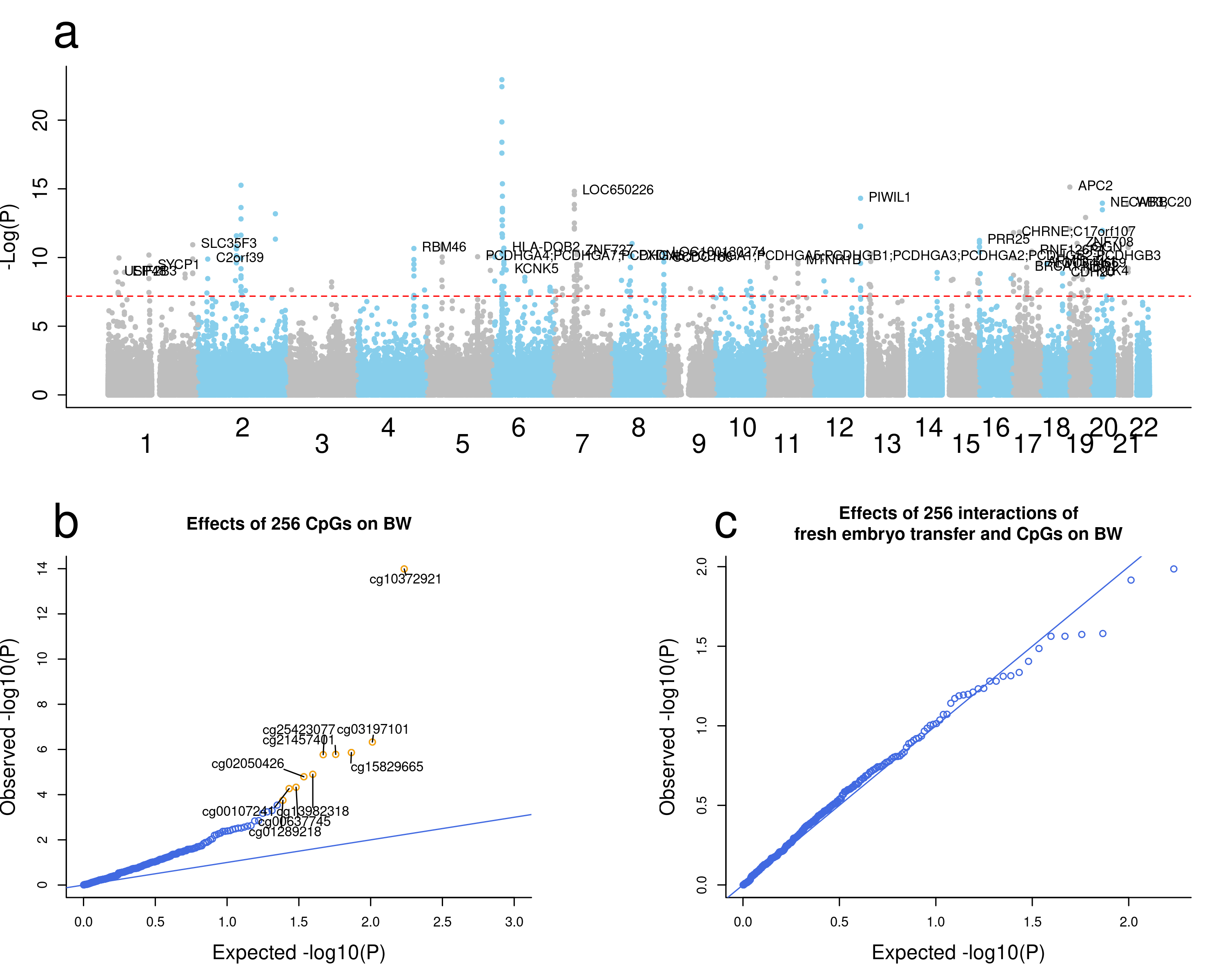

Supplement: Supplementary file 3 — Additional file 3: Fig. S3. Main analysis with additional adjustment for cell-type composition. (a) Manhattan plot displaying the 256 differentially methylated CpGs between newborns conceived naturally and those conceived by fresh embryo transfer. The red dotted line refers to the Bonferroni threshold (P = 0.05/770,564). Adjustment variables include maternal age, smoking status, pre-pregnancy BMI, parity, offspring sex, plate number and cell-type composition. (b) Quantile–quantile plot showing the birthweight-associated CpGs among the 256 fresh embryo transfer-associated CpGs. The yellow dots refer to the CpGs that were also associated with birthweight. Adjustment variables include those mentioned in (a) and maternal education and intake of folic acid. (c) Quantile–quantile plot showing the interactions of fresh embryo transfer and CpGs on birthweight. Adjustment variables were those mentioned in (b). [file 13148_2022_1381_MOESM3_ESM.tiff]

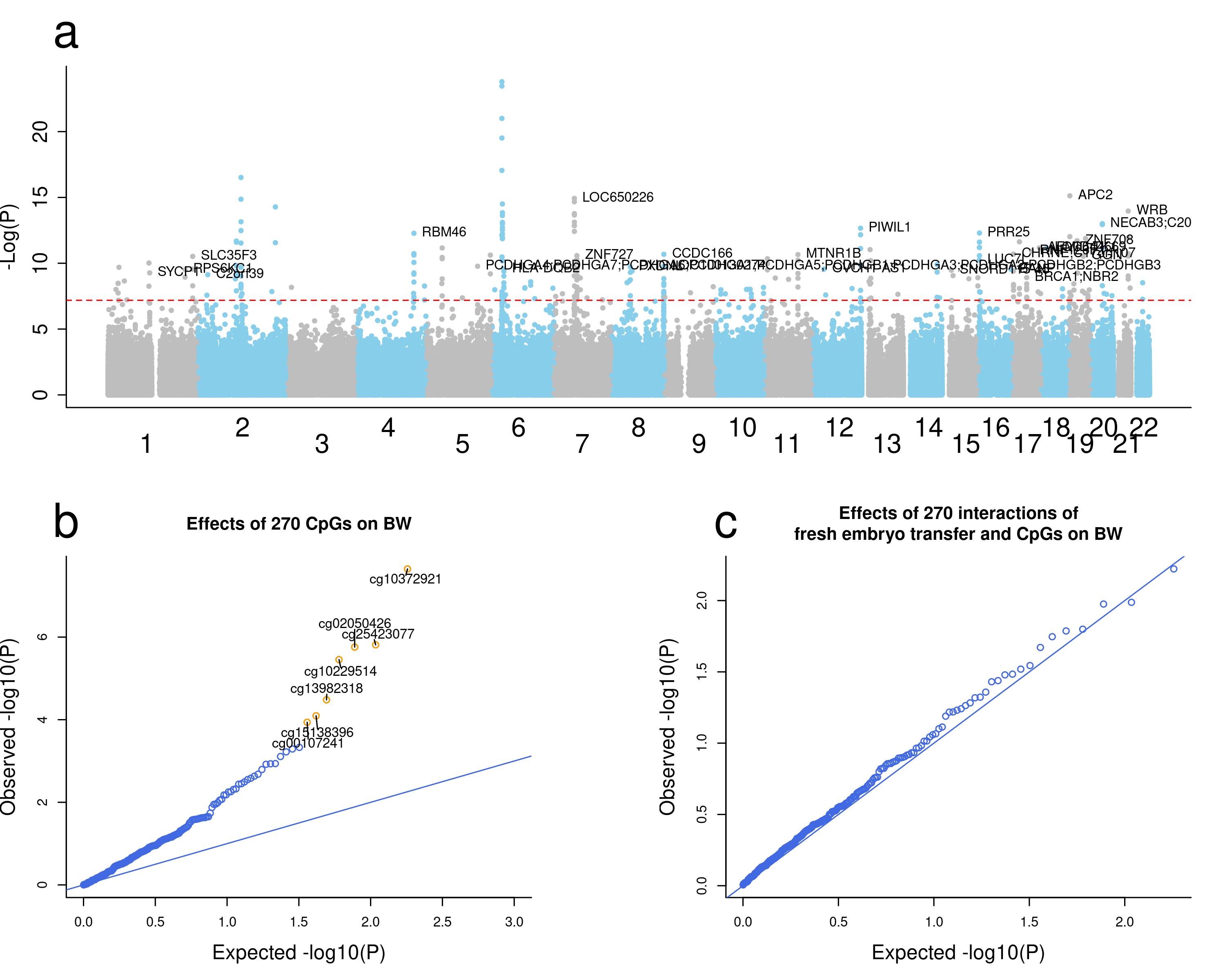

Supplement: Supplementary file 5 — Additional file 5: Fig. S4. Main analysis excluding 20 cases of intrauterine insemination. (a) Manhattan plot displaying the 270 differentially methylated CpGs between newborns conceived naturally (n = 963; the cases of insemination were excluded) and those conceived by fresh embryo transfer (n = 764). The red dotted line refers to the Bonferroni threshold (P = 0.05/770,564). Adjusting variables include maternal age, smoking status, pre-pregnancy BMI, parity, offspring sex, and plate number. (b) Quantile–quantile plot showing the birthweight-associated CpGs among the 270 fresh embryo transfer-associated CpGs. The yellow dots refer to the CpGs that were also associated with birthweight. Adjusting variables include those mentioned in (a) and maternal education and intake of folic acid. (c) Quantile–quantile plot showing the interactions of fresh embryo transfer and CpGs on birthweight. Adjusting variables were those mentioned in (b). [file 13148_2022_1381_MOESM5_ESM.tiff]

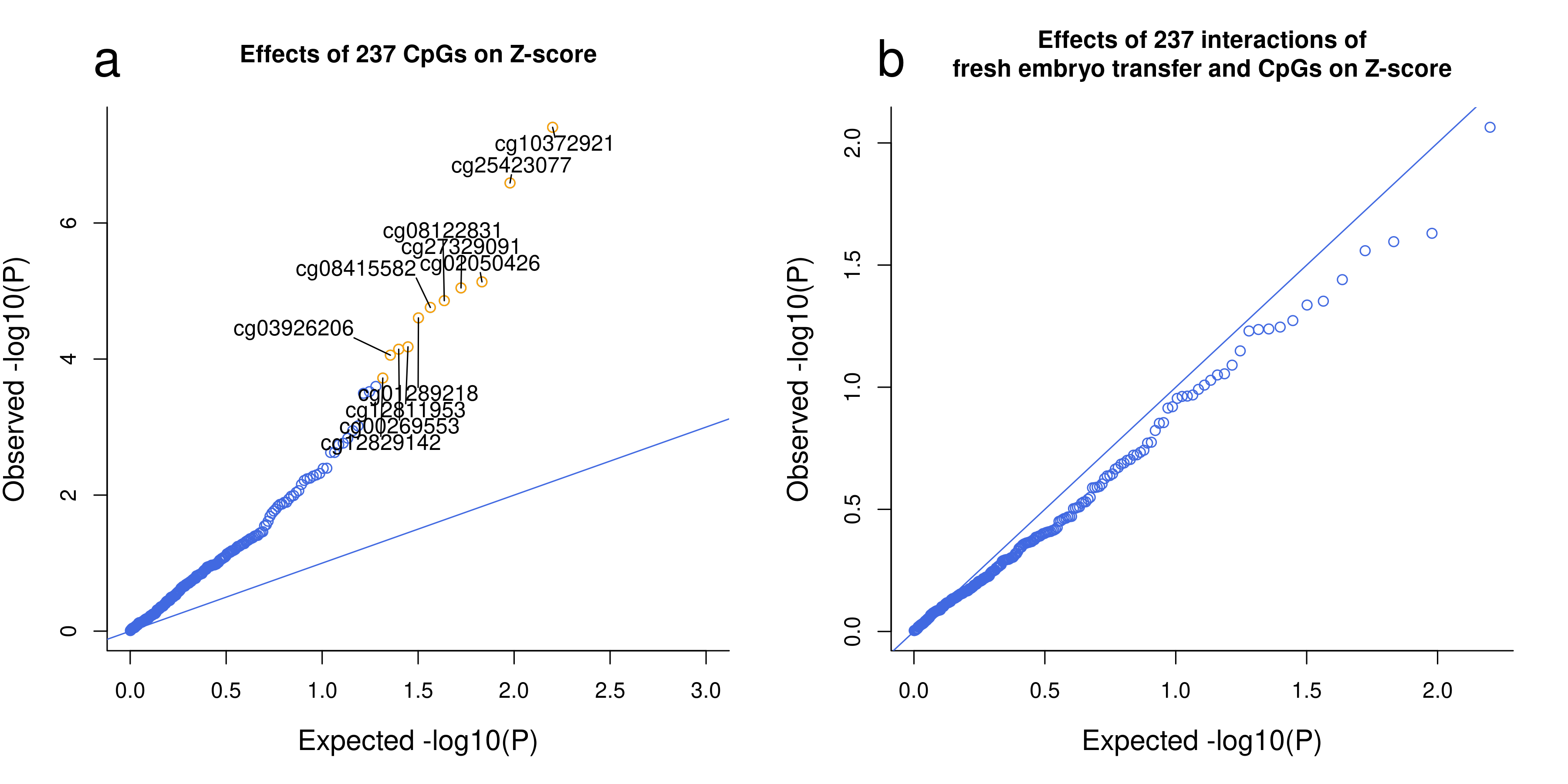

Supplement: Supplementary file 6 — Additional file 6: Fig. S5. Differentially methylated CpGs between newborns conceived naturally and by fresh embryo transfer and Z-score-associated CpGs. (a) Quantile–quantile plot showing the Z-score-associated CpGs among the 237 fresh embryo transfer-associated CpGs. The yellow dots refer to the CpGs that were also associated with Z-score. (b) Quantile–quantile plot showing the interactions of fresh embryo transfer and CpGs on Z-score. [file 13148_2022_1381_MOESM6_ESM.tiff]

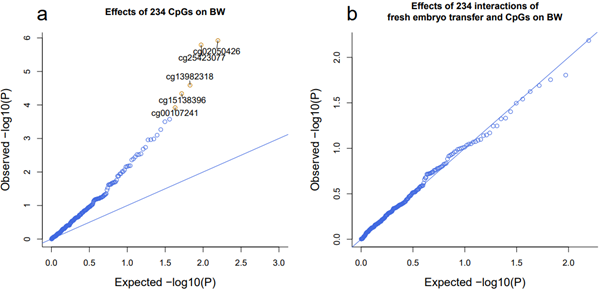

Supplement: Supplementary file 7 — Additional file 7: Fig. S6. Differentially methylated CpGs between newborns conceived naturally and by fresh embryo transfer and birthweight-associated CpGs after applying BACON correction for inflation. (a) Quantile–quantile plot showing the birthweight-associated CpGs among the 234 fresh embryo transfer-associated CpGs. The yellow dots refer to the CpGs that were also associated with birthweight. (b) Quantile–quantile plot showing the interactions of fresh embryo transfer and CpGs on birthweight. [file 13148_2022_1381_MOESM7_ESM.tif]
