# Supplementary material for: An examination of mediation by DNA methylation on birthweight differences induced by assisted reproductive technologies
Source: Clin Epigenetics. 2022 Nov 28;14:151. doi: 10.1186/s13148-022-01381-w (PMC9703677; doi:10.1186/s13148-022-01381-w)
Supplement: Supplementary file 2 — Additional file 2: Fig. S2. Pearson correlation coefficients among the four CpGs mediated between ART (naturally conceived vs fresh embryo transfer) and birthweight. [file 13148_2022_1381_MOESM2_ESM.docx]

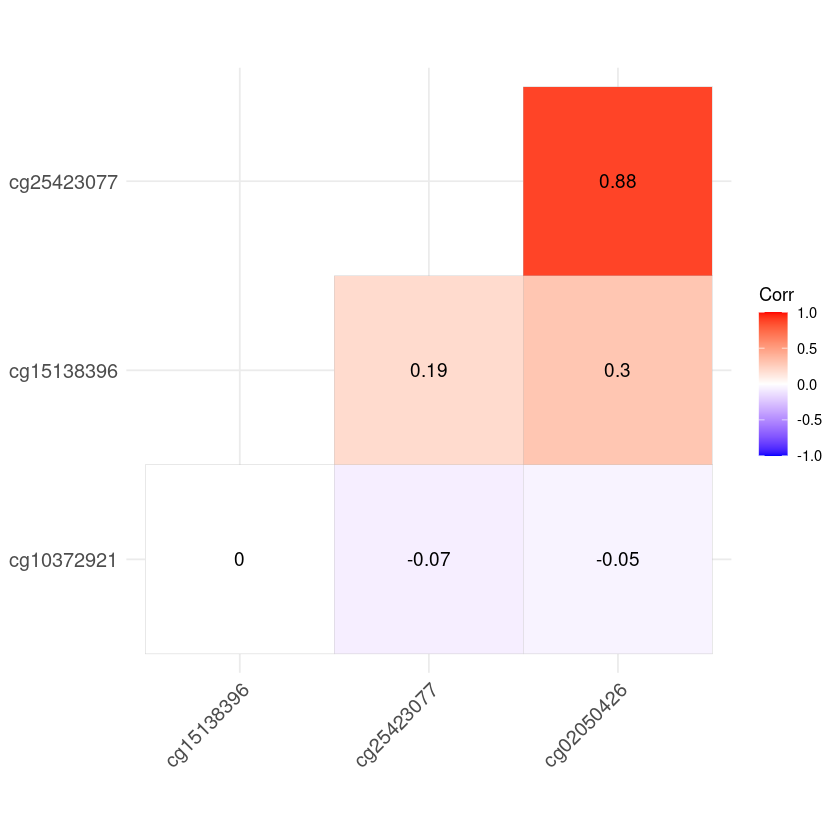


**S-Fig 2. Pearson correlations among the four CpGs that mediated the difference in birthweight between newborns conceived naturally and those conceived by fresh embryo transfer.**
