# Supplementary material for: An examination of mediation by DNA methylation on birthweight differences induced by assisted reproductive technologies
Source: Clin Epigenetics. 2022 Nov 28;14:151. doi: 10.1186/s13148-022-01381-w (PMC9703677; doi:10.1186/s13148-022-01381-w)
Supplement: Supplementary file 4 — Additional file 4: Table S1. Mediation analyses of the birthweight differences between fresh embryo transfer conceived newborns and naturally conceived newborns after additional adjustment for cell-type composition in cord blood. This file contains S-Table 2: mediation analyses of the birthweight differences between fresh embryo transfer conceived newborns and naturally conceived newborns after excluding newborns conceived by intrauterine inseminations from the control group. This file contains S-Table 3: mediation analyses of the growth differences between fresh embryo transfer conceived newborns and naturally conceived newborns now using birthweight Z-score instead of birthweight. This file contains S-Table 4: mediation analyses of the growth differences between fresh embryo transfer conceived newborns and naturally conceived newborns after correction for inflation factor in the EWAS using the BACON method. [file 13148_2022_1381_MOESM4_ESM.docx]

**S-Table 1 Effects of DNA methylation on birthweight differences between fresh and naturally conceived newborns after adjustment for cell-type composition in cord blood.**

|  | **Total effect^1, 2^** | **P-value** |  |  |  |  |  |  |
| --- | --- | --- | --- | --- | --- | --- | --- | --- |
| **Naturally conceived (reference) vs**  **fresh embryo transfer** | -122 (-179, -63) | 3.15E-05 |  |  |  |  |  |  |
|  |  |  |  |  |  |  |  |  |
| **CpG name** | **Indirect effect^1, 2^ in grams** | **P-value^1^** | **Direct effect^1, 2^ in grams** | **P-value** | **Mediation  proportion** | **CHR^3^** | **MAPINFO^3^** | **UCSC**  **RefGene**  **Name^3^** |
| cg10372921 | -30 (-44, -19) | 2.80E-06 | -92 (-149, -34) | 1.67E-03 | 26.6% | 15 | 74218733 | *LOXL1^4^* |
| cg03197101 | -17 (-28, -8) | 9.21E-04 | -105 (-163, -48) | 3.57E-04 | 14.6% | 6 | 160241521 | *PNLDC1*^5^ |
| cg25423077 | -17 (-27, -7) | 1.03E-03 | -105 (-164, -47) | 3.57E-04 | 14.6% | 18 | 59221601 | *CDH20*^5^ |
| cg21457401 | -16 (-26, -7) | 1.12E-03 | -106 (-164, -48) | 3.12E-04 | 13.8% | 6 | 160241519 | *PNLDC1*^5^ |
| cg15829665 | -16 (-26, -7) | 1.25E-03 | -106 (-164, -48) | 3.25E-04 | 14.1% | 6 | 160241556 | *PNLDC1^6^* |
| cg13982318 | -14 (-24, -6) | 2.68E-03 | -108 (-165, -50) | 2.38E-04 | 12.3% | 9 | 72131655 | *APBA1*^5^ |
| cg02050426 | -13 (-23, -5) | 3.23E-03 | -108 (-166, -50) | 2.35E-04 | 11.9% | 18 | 59221458 | *CDH20*^5^ |
| cg00637745 | -14 (-24, -5) | 3.78E-03 | -108 (-166, -50) | 2.56E-04 | 12.1% | 2 | 121497334 |  |
| cg01289218 | -12 (-21, -4) | 9.13E-03 | -110 (-168, -52) | 1.97E-04 | 10.3% | 4 | 155702636 | *RBM46^7^* |
| cg00107241 | -12 (-22, -4) | 1.02E-02 | -110 (-168, -52) | 1.89E-04 | 10.3% | 1 | 29461070 |  |
|  |  |  |  |  |  |  |  |  |
|  | **Combined**  **indirect effect^1, 2^** | **P-value^1^** | **Combined**  **direct effect^1, 2^** | **P-value** | **Mediation  proportion** |  |  |  |
| **The ten CpGs**  **combined** | -67 (-87, -47) | 4.82E-11 | -55 (-114, 3) | 6.22E-02 | 54.7% |  |  |  |

^1^ Adjusted for maternal age, education, smoking status, pre-pregnancy BMI, parity, child’s sex, maternal intake of folic acid supplement, cord blood cell type composition, and plate number. Birthweight difference is in grams.

^2^ 95% confidence intervals in parenthesis.

^3^ Derived from the Illumina MethylationEPIC v1.0 B5 manifest file. The archaic names of the genes were updated based on the data from <https://www.genenames.org/>.

^4^ Located within 0-200 bases upstream of the transcription start site of the gene.

^5^ Located within the gene body.

^6^ Located within the 3’ untranslated region of the gene.

^7^ Located within 0-200 bases upstream of the transcription start site of the gene or within the 5’ untranslated region.

**S-Table 2 Effects of ART on birthweight mediated through DNA methylation in newborns conceived by fresh embryo transfer compared to those conceived naturally after excluding 20 cases of intrauterine insemination.**

|  | Total effect^1, 2^ | P-value^2^ |  |  |  |  |  |  |
| --- | --- | --- | --- | --- | --- | --- | --- | --- |
| **Naturally conceived (reference) vs**  **fresh embryo transfer** | -129 (-188, -70) | 2.34E-05 |  |  |  |  |  |  |
|  |  |  |  |  |  |  |  |  |
| CpG name | Indirect effect^1, 2^ in grams | P-value^2^ | Direct effect^1, 2^ in grams | P-value^2^ | Mediation  proportion | CHR^3^ | MAPINFO^3^ | UCSC  RefGene  Name^3^ |
| cg10372921 | -24 (-36,-13) | 4.17E-05 | -105 (-164, -45) | 5.72E-04 | 19.8% | 15 | 74218733 | *LOXL1^4^* |
| cg10229514 | -18 (-29,-8) | 9.76E-04 | -111 (-171, -51) | 3.56E-04 | 14.9% | 7 | 134104798 |  |
| cg25423077 | -16 (-27,-7) | 1.24E-03 | -113 (-172, -54) | 2.14E-04 | 13.2% | 18 | 59221601 | *CDH20^5^* |
| cg02050426 | -15 (-25,-7) | 1.64E-03 | -114 (-173, -55) | 1.86E-04 | 12.2% | 18 | 59221458 | *CDH20^5^* |
| cg13982318 | -13 (-24,-5) | 4.41E-03 | -115 (-175, -57) | 1.53E-04 | 11.1% | 9 | 72131655 | *APBA1^5^* |
| cg15138396 | -15 (-26,-5) | 5.80E-03 | -114 (-175, -53) | 2.43E-04 | 12.2% | 2 | 26624450 | *DRC1^6^* |
| cg00107241 | -11 (-21,-3) | 1.51E-02 | -118 (-177, -58) | 1.19E-04 | 9.1% | 1 | 29461070 |  |
|  |  |  |  |  |  |  |  |  |
|  | Combined  indirect effect^1, 2^ | P-value^2^ | Combined  direct effect^1, 2^ | P-value^2^ | Mediation  proportion |  |  |  |
| The seven CpGs combined | -83 (-106, -61) | 1.41E-12 | -46 (-107, 16) | 1.47E-01 | 64.3% |  |  |  |

^1^ 95% confidence interval in parentheses.

^2^ Adjusted for maternal age, education, smoking status, pre-pregnancy BMI, parity, child’s sex, maternal intake of folic acid supplement, and plate number.

^3^ Derived from the Illumina MethylationEPIC v1.0 B5 manifest file. The archaic names of the genes were updated based on the data from <https://www.genenames.org/>.

^4^ Located within 0-200 bases upstream of the transcription start site of the gene.

^5^ Located within the gene body.

^6^ Located within 200-1500 bases upstream of the transcription start site of the gene.

**S-Table 3 Effects of the use of ART procedure on Z-score mediated through DNA methylation in newborns conceived naturally compared to those conceived by fresh embryo transfer.**

|  | **Total effect^1^** | **Sobel P** |  |  |  |  |  |  |
| --- | --- | --- | --- | --- | --- | --- | --- | --- |
| **Naturally conceived (reference) vs**  **fresh embryo transfer** | -0.22 (-0.33, -0.11) | 1.47E-04 |  |  |  |  |  |  |
|  |  |  |  |  |  |  |  |  |
| **CpG name** | **Indirect effect^1, 2^ in grams** | **Sobel P^2^** | **Direct effect^1^ in grams** | **P-value** | **Mediation  proportion** | **CHR^3^** | **MAPINFO^3^** | **UCSC**  **RefGene**  **Name^3^** |
| cg10372921 | -0.04 (-0.07, -0.03) | 3.24E-05 | -0.17 (-0.29, -0.06) | 2.37E-03 | 20.7% | 15 | 74218733 | *LOXL1^4^* |
| cg25423077 | -0.03 (-0.05, -0.02) | 6.79E-04 | -0.18 (-0.3, -0.07) | 1.33E-03 | 16.2% | 18 | 59221601 | *CDH20^5^* |
| cg08122831 | -0.04 (-0.06, -0.02) | 1.10E-03 | -0.18 (-0.29, -0.06) | 1.89E-03 | 18.1% | 2 | 213697579 |  |
| cg27329091 | -0.04 (-0.06, -0.02) | 1.10E-03 | -0.18 (-0.3, -0.07) | 1.72E-03 | 17.0% | 2 | 213698049 |  |
| cg12829142 | 0.04 (0.02, 0.06) | 1.19E-03 | -0.25 (-0.37, -0.14) | 1.32E-05 | -16.5% | 7 | 23529999 | *RPS2P32^4^* |
| cg08415582 | -0.03 (-0.05, -0.01) | 1.52E-03 | -0.19 (-0.3, -0.07) | 1.38E-03 | 14.7% | 8 | 57030523 |  |
| cg02050426 | -0.03 (-0.05, -0.01) | 2.56E-03 | -0.19 (-0.3, -0.08) | 8.89E-04 | 12.6% | 18 | 59221458 | *CDH20^5^* |
| cg01289218 | -0.03 (-0.04, -0.01) | 3.27E-03 | -0.19 (-0.3, -0.08) | 8.59E-04 | 12.1% | 4 | 155702636 | *RBM46^6^* |
| cg03926206 | -0.03 (-0.05, -0.01) | 4.60E-03 | -0.19 (-0.3, -0.08) | 1.07E-03 | 13.8% | 6 | 32729358 | *HLA-DQB^5^* |
| cg00269553 | -0.03 (-0.05, -0.01) | 5.36E-03 | -0.19 (-0.3, -0.08) | 1.17E-03 | 13.7% | 8 | 52321814 | *PXDNL^5^* |
| cg12811953 | -0.02 (-0.04, -0.01) | 6.16E-03 | -0.19 (-0.31, -0.08) | 8.57E-04 | 10.6% | 1 | 22111043 | *USP48^7^* |
|  |  |  |  |  |  |  |  |  |
|  | **Combined**  **indirect effect^1, 2^** | **Sobel P^2^** | **Combined**  **direct effect^1^** | **Sobel P** | **Mediation  proportion** |  |  |  |
| **The eleven CpGs combined** | -0.07 (-0.12, -0.03) | 8.29E-04 | -0.14 (-0.26, -0.03) | 1.68E-02 | 34.2% |  |  |  |

^1^ 95% confidence interval in parentheses.

^2^ Adjusted for maternal age, education, smoking, pre-pregnancy BMI, parity, child’s sex, maternal intake of folic acid supplement, and plate number.

^3^ Derived from the Illumina MethylationEPIC v1.0 B5 manifest file. The old names of the genes were updated based on the data from <https://www.genenames.org/>.

^4^ Located within 0-200 bases upstream of the transcription start site of the gene.

^5^ Located within the gene body.

^6^ Located within 0-200 bases upstream of the transcription start site of the gene or within the 5’ untranslated region.

^7^ Located within 200-1500 bases upstream of the transcription start site of the gene.

**S-Table 4 Effects of DNA methylation on birthweight differences between fresh and naturally conceived newborns after correction for the inflation factor applying the BACON method.**

|  | **Total effect^1^** | **Sobel P** |  |  |  |  |  |  |
| --- | --- | --- | --- | --- | --- | --- | --- | --- |
| **Naturally conceived (reference) vs**  **fresh embryo transfer** | -120 (-179, -61) | 6.35E-05 |  |  |  |  |  |  |
|  |  |  |  |  |  |  |  |  |
| **CpG name** | **Indirect effect^1,2^ in grams** | **P-value^2^** | **Direct effect^1,2^ in grams** | **P-value** | **Mediation  proportion** | **CHR^3^** | **MAPINFO^3^** | **UCSC RefGene Name^3^** |
| cg25423077 | -16 (-27, -8) | 1.04E-03 | -104 (-163, -45) | 5.85E-04 | 14.5% | 18 | 59221601 | *CDH20^4^* |
| cg02050426 | -15 (-26, -7) | 1.41E-03 | -105 (-164, -46) | 4.94E-04 | 13.5% | 18 | 59221458 | *CDH20^4^* |
| cg13982318 | -13 (-22, -5) | 3.55E-03 | -108 (-166, -49) | 3.37E-04 | 11.4% | 9 | 72131655 | *APBA1^4^* |
| cg15138396 | -15 (-27, -6) | 3.98E-03 | -105 (-165, -44) | 5.86E-04 | 13.6% | 2 | 26624450 | *DRC1^5^* |
| cg00107241 | -11 (-21, -3) | 1.54E-02 | -109 (-168, -49) | 3.04E-04 | 9.8% | 1 | 29461070 |  |
|  |  |  |  |  |  |  |  |  |
|  | **Combined indirect effect^1,2^** | **P-value^2^** | **Combined direct effect^1,2^** | **P-value** | **Mediation  proportion** |  |  |  |
| The CpGs combined | -34 (-50, -20) | 8.89E-06 | -86 (-145, -25) | 5.08E-03 | 28.4% |  |  |  |

^1^ 95% confidence intervals in parenthesis.

^2^ Adjusted for maternal age, education, smoking status, pre-pregnancy BMI, parity, child’s sex, maternal intake of folic acid, and plate number. Birthweight difference is in grams.

^3^ Derived from the Illumina MethylationEPIC v1.0 B5 manifest file. The archaic names of the genes were updated based on the data from <https://www.genenames.org/>.

^4^ Located within the gene body.

^5^ Located within 200-1500 bases upstream of the transcription start site of the gene.
